# Analysis of clonal expansions through the normal and premalignant human breast epithelium reveals the presence of luminal stem cells

**DOI:** 10.1002/path.4989

**Published:** 2017-11-23

**Authors:** Biancastella Cereser, Marnix Jansen, Emily Austin, George Elia, Taneisha McFarlane, Carolien HM van Deurzen, Anieta M Sieuwerts, Maria G Daidone, Paul J Tadrous, Nicholas A Wright, Louise Jones, Stuart AC McDonald

**Affiliations:** ^1^ Clonal Dynamics in Epithelia Laboratory, Centre for Tumour Biology Barts Cancer Institute, Queen Mary University of London UK; ^2^ Epithelial Stem Cell Laboratory, Centre for Tumour Biology Barts Cancer Institute, Queen Mary University of London UK; ^3^ Centre for Histopathology Laboratory, Barts Cancer Institute Queen Mary University of London UK; ^4^ Department of Surgery and Cancer, Imperial College London Charing Cross Hospital London UK; ^5^ Department of Pathology, Erasmus MC Cancer Institute Erasmus University Medical Center Rotterdam The Netherlands; ^6^ Department of Medical Oncology, Erasmus MC Cancer Institute Erasmus University Medical Center, Rotterdam The Netherlands; ^7^ Department of Experimental Oncology and Molecular Medicine Fondazione IRCCS Istituto Nazionale dei Tumori Milan Italy; ^8^ Department of Cellular Pathology Northwick Park Hospital London UK; ^9^ Breast Cancer Laboratory, Centre for Tumour Biology Barts Cancer Institute, Queen Mary University of London UK

**Keywords:** clonality, stem cells, mammary stem cell niche, ductal carcinoma in situ

## Abstract

It is widely accepted that the cell of origin of breast cancer is the adult mammary epithelial stem cell; however, demonstrating the presence and location of tissue stem cells in the human breast has proved difficult. Furthermore, we do not know the clonal architecture of the normal and premalignant mammary epithelium or its cellular hierarchy. Here, we use deficiency in the mitochondrial enzyme cytochrome c oxidase (CCO), typically caused by somatic mutations in the mitochondrial genome, as a means to perform lineage tracing in the human mammary epithelium. PCR sequencing of laser‐capture microdissected cells in combination with immunohistochemistry for markers of lineage differentiation was performed to determine the clonal nature of the mammary epithelium. We have shown that in the normal human breast, clonal expansions (defined here by areas of CCO deficiency) are typically uncommon and of limited size, but can occur at any site within the adult mammary epithelium. The presence of a stem cell population was shown by demonstrating multi‐lineage differentiation within CCO‐deficient areas. Interestingly, we observed infrequent CCO deficiency that was restricted to luminal cells, suggesting that niche succession, and by inference stem cell location, is located within the luminal layer. CCO‐deficient areas appeared large within areas of ductal carcinoma in situ, suggesting that the rate of clonal expansion was altered in the premalignant lesion. © 2017 The Authors. *The Journal of Pathology* published by John Wiley & Sons Ltd on behalf of Pathological Society of Great Britain and Ireland.

## Introduction

The stem cell hierarchy of the human mammary epithelium has been the subject of much debate. Previous studies suggest that stem cells in the normal human breast are located within the luminal epithelial layer and also give rise to the myoepithelial cells [1, 2]. However, *in vitro* studies show the possible existence of progenitor cells that may differentiate into luminal cells from either the myoepithelial or the luminal lineages, or indeed from both [1, 2, 3, 4, 5, 6, 7]. There is further evidence for a subset of luminal cells that express cytokeratin 5 (CK5) and can give rise to both luminal and myoepithelial lineages. This subset may also represent a stem cell population and potentially act as cells of origin for breast cancer [8, 9, 10]. In addition, a recent study in human tissue combining a novel 3D fractal model approach with a theoretical model and with the expression of the putative stem cell marker high aldehyde dehydrogenase (ALDH1A1) has suggested that during morphogenesis of the mammary gland, the intralobular branching ducts are the site of cellular expansion and growth. This would indicate that this site may be the location of stem cells within the adult breast 11. However, a novel analysis of multicolour lineage tracing at saturation during pubertal development of the mouse mammary gland rules out the presence and role of multipotent stem cells during adult tissue remodelling 12.

Consequently, the location and characterization of stem cells in the human breast are still unknown. The major hindrance to our understanding of the location of the human breast stem cell has been a lack of markers that definitively demonstrate multi‐lineage differentiation and clonal expansion within tissue sections. To date, no human lineage tracing studies have been performed to show this. To determine the location of stem cells within the human mammary epithelium, we have used a lineage tracing technique where mitochondrial DNA (mtDNA) mutations act as a marker of clonal expansion 13. Mutant cells are identified by the deficiency of the mitochondrial enzyme cytochrome *c* oxidase (CCO). Serial sections subjected to immunohistochemistry for lineage‐specific markers, in combination with sequencing of the mitochondrial genome from distinct microdissected mammary epithelial cells, demonstrated multi‐lineage differentiation, which is the gold standard for stem cell identification 14. MtDNA mutations accumulate within normal tissue stem cells and increase in frequency with age, reaching homoplasmy or detectable levels of heteroplasmy in mid to late life 15. We have shown previously that this method allows identification of the stem cell niche in the human stomach 16, small bowel 17, and normal and premalignant prostate 18. Furthermore, somatic mtDNA mutations are neutral, conferring no selective advantage or disadvantage permitting analysis of steady‐state clonal competition within the normal human mammary gland 19.

Here, we investigate in detail the clonal architecture of the normal and premalignant epithelium *in situ* in the human mammary gland. Stem cells have been long considered the likely origin of cancer [20, 21]; therefore, our findings may shine light not only on homeostasis of the normal mammary gland but also on their contribution to the origin of premalignant lesions and invasive cancer. Here, we show that clonal expansions demonstrating multi‐lineage differentiation from a single stem cell can occur in any area of the normal human breast epithelium.

Ductal carcinoma *in situ* (DCIS) is considered unanimously to be a precursor of invasive ductal cancer (IDC), because several studies have found a link between genetic alterations which occur in the premalignant lesion and are maintained in the invasive lesion [22, 23, 24, 25].

However, the human DCIS stem cell has not been identified, nor has the extent to which a stem cell's progeny can expand through the breast. Here, we show lineage tracing within human DCIS that may provide an insight into its cell of origin, a mode of expansion within the human breast, and a potential understanding of the neoplastic process.

## Materials and methods

### Tissue

Fresh‐frozen and formalin‐fixed, paraffin‐embedded (FFPE) clinical samples from patients undergoing breast surgery between 2004 and 2009 at Barts Health NHS Trust, London, UK were studied following patient consent and approval from the local research ethics committee and deposited in the Breast Cancer Now tissue bank (formerly Breast Cancer Campaign tissue bank; ref: 10/H0308/49). Fresh‐frozen DCIS clinical samples were also obtained from the Erasmus Medical Center Rotterdam, The Netherlands (MEC 02.953), with the study adhering to the Code of Conduct of the Federation of Medical Scientific Societies in The Netherlands; from the Imperial College London Tissue Bank, UK, following patient consent and approval from the local research ethics committee (ref: ICHTB HTA; licence: 12275; REC Wales approval: 12/WA/0196); and from the Fondazione IRCSS, Istituto Nazionale Tumori, Milano, Italy (ref: INT 199/15).

### Enzyme histochemistry

Frozen sections (16 μm) of breast tissue underwent sequential CCO and succinate dehydrogenase (SDH) enzyme histochemistry, as described previously 15. CCO/SDH histochemistry permits the detection of CCO‐normal cells (brown) contrasting with CCO‐deficient cells (blue due to SDH activity). In brief, sections were incubated first in cytochrome *c* medium [100 mm cytochrome *c*, 4 mm diaminobenzidine tetrahydrochloride (brown chromogen), 20 μg/ml catalase in 0.2 m phosphate buffer, pH 7.0; all from Sigma Aldrich, Poole, UK] for 40 min at 37 °C to allow detection of CCO activity in brown, followed by washes in PBS, pH 7.4, for 3 × 5 min and then by incubation in SDH medium (130 mm sodium succinate, 200 mm phenazine methosulphate, 1 mm sodium azide, 1.5 mm nitroblue tetrazolium in 0.2 m phosphate buffer, pH 7.0) for 45 min at 37 °C to allow detection of SDH activity with nitroblue tetrazolium (blue chromogen). Sections were allowed to dry in air for microdissection or dehydrated in increasing ethanol concentrations followed by clearing in Histoclear (Fisher Scientific, Leicestershire, UK). All images were captured using a Pannoramic 250 Flash III scanner and viewed using Pannoramic viewer software (3D Histotech, Budapest, Hungary).

### Immunohistochemistry

Five‐micrometre‐thick FFPE tissue sections were dewaxed and subjected to boiling in 10 mm sodium citrate buffer solution, pH 6.0 (Sigma, UK) for 20 min. Endogenous peroxidase activity was blocked with 3% hydrogen peroxide solution for 10 min, followed by a serum‐free protein block (Dako, Ely, UK) for 10 min. Sections were incubated for 1 h at room temperature with primary antibody mouse anti‐human CCO (OxPhos Complex IV subunit I, 106E1A8; Life Technologies, Paisley, UK) at a 1:100 dilution in blocking serum, followed by incubation for 40 min at room temperature in biotin‐conjugated goat anti‐mouse IgG (1:500; Dako). Sections were then incubated in streptavidin‐conjugated HRP (1:500; Dako) for 30 min at room temperature. Colour was developed with a DAB Peroxidase (HRP) Substrate Kit (Vector Laboratories, Peterborough, UK) according to the manufacturer's recommendations and counterstained with haematoxylin, before dehydration through alcohol, clearing in xylene, and mounting.

### Fluorescence immunohistochemistry

FFPE tissue sections were dewaxed and unmasked as above. Fresh‐frozen sections were fixed in an ice‐cold 1:1 acetone–methanol solution for 5 min at room temperature. Sections were blocked with serum‐free protein block (Dako) for 40 min. Sections were then incubated for 1 h at room temperature with the primary antibodies αSMA (1A4; Dako), CK18 (EPR1626; Abcam, Cambridge, UK), both at a 1:50 dilution in blocking serum, and mouse anti‐CCO (OxPhos Complex IV subunit I; 1D6‐E1A8; Life Technologies) at a 1:100 dilution in blocking serum, followed by incubation for 40 min at room temperature in Alexa Fluor 488‐conjugated goat anti‐mouse IgG or Alexa Fluor 594‐conjugated goat anti‐rabbit IgG (Life Technologies) added at a 1:1500 dilution in blocking serum. Sections were mounted in Prolong Gold anti‐fade with DAPI (Invitrogen, Carlsbad, CA, USA) and analysed using an Axioplan microscope equipped with AxioCam MRc and AxioVision software (Zeiss, Munich, Germany). In each analysis, positive and negative controls were available. When enzyme histochemistry was combined with IHC on the same section, CCO histochemistry was performed as described first, followed by fixation with a 1:1 acetone–methanol solution as above.

### Extraction of mtDNA from microdissected tissue

Frozen sections (16 μm thick) were cut onto PALM membrane slides (Zeiss) and air‐dried at room temperature for 1 h and then subjected to enzymatic CCO staining as described above. Single cells or larger areas of interest from mammary ducts and terminal duct lobular units (TDLUs) were then microdissected on a PALM laser capture system (Zeiss) at a uniform laser power and cutting width into PALM‐specific 0.5 ml tubes. Stromal tissue was used as a control from each section. DNA was extracted using QIAamp DNA Micro kits (Qiagen, Hilden, Germany) according to the manufacturer's protocol.

### Sanger sequencing

A nested PCR protocol producing 36 500‐bp overlapping fragments covering the entire mitochondrial genome (mtDNA) was followed as described previously 15. PCR products were treated with ExoSaP‐IT (GE Healthcare, Little Chalfont, UK) according to the manufacturer's protocol and subjected to a Sanger sequencing reaction using BigDye 3.1 (Life Technologies) and then purified by ethanol precipitation and run on an ABI Prism 3100 genetic analyzer (Life Technologies). Sequence traces were analysed using 4Peaks software (http://nucleobytes.com/; formerly mekentosj.com) together with Clustal W2 software (EMBL‐EBI) and compared with the revised Cambridge reference sequence 26 and sequences from stromal controls and CCO‐normal specimens to eliminate polymorphisms from the CCO‐deficient sequences.

## Results

### Visualization of clonal expansions within normal and pre‐invasive (DCIS) human mammary epithelium

**Figure 1:**
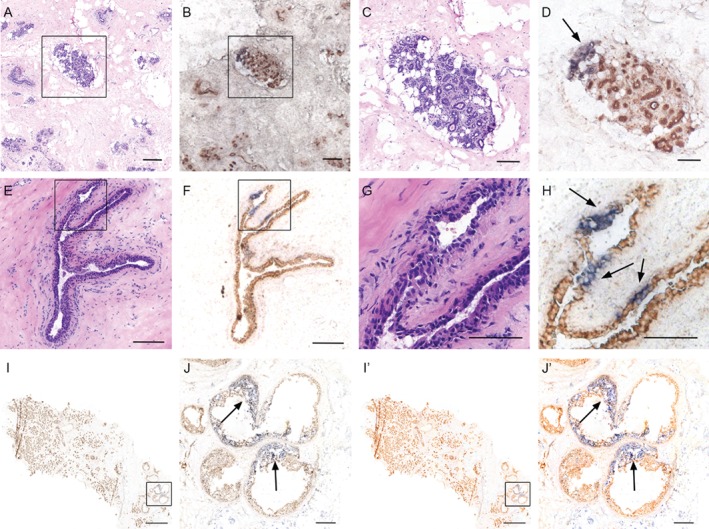
CCO‐deficient patches of cells are found through the normal and premalignant human breast. (A) H&E staining showing a TDLU in the normal adult breast. (B) CCO enzyme histochemistry identifies a subset of cells within the TDLU containing blue, CCO‐deficient cells. High‐power images are shown in C and D, respectively. CCO deficiency is indicated by arrows. CCO‐deficient ducts are also found in the ducts of normal human breast. (E) H&E staining showing a normal duct from adult human breast. (F) CCO enzyme histochemistry identifies three similarly distinct clusters of cells within the normal duct containing blue, CCO‐deficient cells. High‐power images are shown in G and H, respectively. Scale bar = 150 μm; inset scale bar = 75 μm. (I) (and outlined area in J) CCO enzyme histochemistry of a sample of invasive breast cancer with adjacent areas of DCIS identifies a large area of CCO‐deficient blue cells within the premalignant lesion. CCO‐deficient cells are interspersed with wild‐type CCO‐positive brown cells, indicating dynamic mixing of clones in DCIS. Scale bar (I) = 2000 μm; inset scale bar (J) = 250 μm. I' and J' represent globally saturated images (saturation set to 60) to highlight the CCO‐deficient areas in I and J, respectively.

To determine the presence of putative progenitor/stem cells in the human breast, we first performed dual enzyme histochemistry for CCO activity (brown) and succinate dehydrogenase (blue, to highlight CCO deficiency), which has been shown previously to highlight clonally related cells 27. We detected areas of CCO deficiency in 9/45 patients' (20%) normal breast specimens and in 5/54 (9.2%) DCIS patients. CCO deficiency in the normal breast was limited to small epithelial patches. These CCO‐deficient patches were detected in terminal duct lobular units (TDLUs) (Figure 1A–D) as well as in ducts (Figure 1E–H). CCO‐deficient areas were also detected in areas of DCIS, but were not detected as frequently as in the normal breast. However, CCO‐deficient areas in DCIS appeared to be larger in size, covering either part of or the entire cross‐section of the lesion (Figure 1I, J).

**Figure 2:**
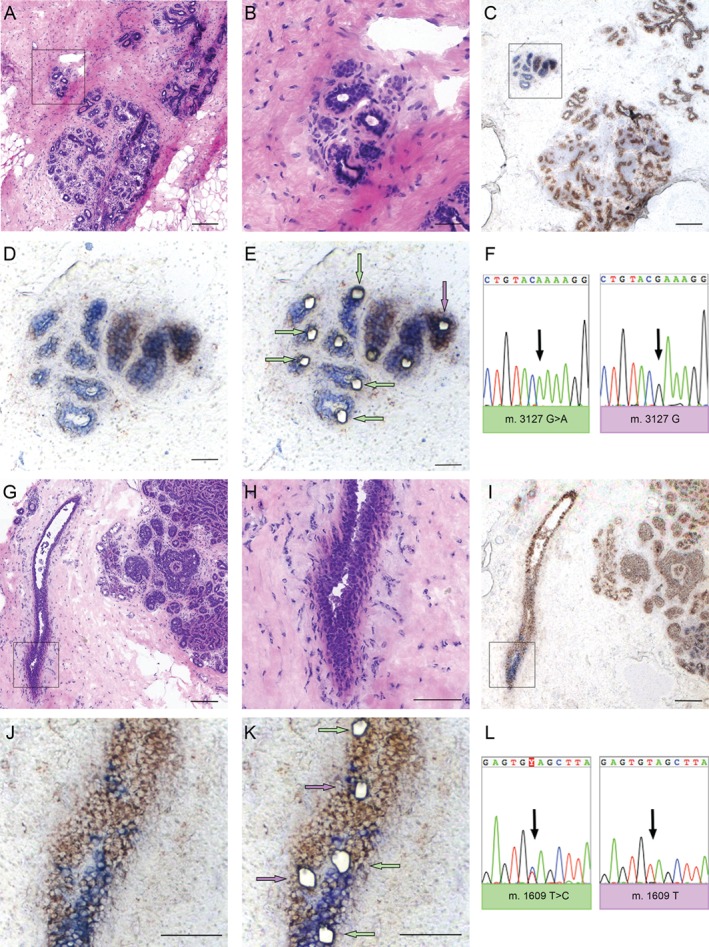
Clonal expansions occur in both TDLUs and ducts. (A) H&E staining showing a TDLU in a normal adult breast and (B) at higher‐power magnification. (C) CCO enzyme histochemistry identifies a discrete TDLU containing blue, CCO‐deficient cells and (D) at higher magnification. (E) Post‐laser capture microdissection of single cells from multiple CCO‐deficient blue areas (arrowed in green) together with adjacent CCO‐normal brown cells (arrowed in purple, and one cell at greater distance in the section, not shown). Those cells without arrows failed to PCR amplify. (F) All CCO‐deficient cells shared a common, clonal point mutation (3127G > A) that was not present in the control CCO‐normal cells. This demonstrated clonal expansion within a TDLU. CCO‐deficient ducts also show clonal expansions. (G) H&E staining showing a normal duct in proximity to DCIS and (H) in higher magnification. (I) CCO enzyme histochemistry identifies clusters of blue, CCO‐deficient cells seen at higher‐power magnification (J) pre‐ and (K) post‐laser microdissection. (L) MtDNA sequencing of single cells from multiple blue cells (arrowed in K) versus brown wild‐type cells from a distant area (not shown) demonstrated that two blue cells from the larger blue cluster (arrowed in green in K) shared a common, heteroplasmic 1609 T > C mutation that was also present in the single cell laser‐captured from a similarly distinct area (arrowed in green at the top of the image) but was not present in adjacent brown cells or in the other cells laser‐captured from distinct blue areas (arrowed in purple). These findings showed that normal ducts are clonal and that multiple clones compete for the monoclonal conversion of the entire duct. Scale bar = 150 μm; inset scale bar = 75 μm.

**Figure 3:**
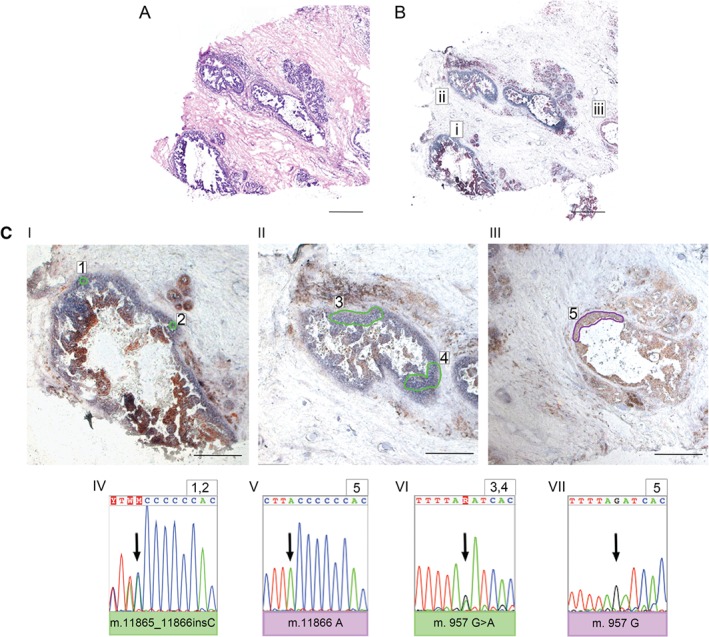
DCIS ducts show multiple, large clonal expansions. (A) H&E staining showing adult breast with DCIS ducts. (B, Ci–iii) CCO histochemistry on the serial section showing both CCO‐deficient (Ci, ii) and CCO‐proficient (Ciii) DCIS ducts. Areas 1 and 2 from Ci show a shared 11867_118673insG mutation (shown in the reverse sequence strand) that was not detected in area 5 (Ciii). The adjacent CCO‐deficient duct (Cii, areas 3 and 4) did not share the same mutation but was clonal for a heteroplasmic 957G > A mutation that was also not detected in area 5 (Ciii). Representative Sanger sequencing traces are shown below Ci–iii. Scale bar = 600 μm; inset scale bar = 300 μm.

To formally demonstrate that patches of CCO‐deficient cells represent bona fide clonal expansions, multiple CCO‐deficient (blue) cells from both normal and DCIS cases were non‐contact laser‐capture microdissected and their entire mtDNA genome was sequenced to reveal common mutations that would indicate a common cell of origin. Figure 2 shows a TLDU that possessed both CCO‐deficient and CCO‐normal cells (Figure 2A–E). All microdissected cells from CCO‐deficient areas contained the same mtDNA mutation (3127G > A), which was not present in the surrounding CCO‐proficient cells, demonstrating a clonal expansion (Figure 2F). Clonal expansions were also observed in CCO‐deficient areas in ducts. Figure 3 shows a classical duct (Figure 2G, H) that contained a small CCO‐deficient area (Figure 2I–K) and each deficient cell harboured a 1609 T > C mtDNA mutation (Figure 2L). These data suggest that clonal expansions may arise within the normal human breast in both ductal and TDLU epithelium.

In DCIS, CCO‐deficient areas appeared much larger than in normal breast epithelium: entire DCIS ducts were clonal, each area containing a clonal mtDNA mutation. Figure 3A shows an H&E‐stained section of an area of DCIS and Figure 3B shows the same area stained for CCO activity. CCO‐deficient DCIS ducts (Figure 3Bi, ii) and a CCO‐proficient duct (Figure 3Biii) were present. Distinct areas microdissected from CCO‐deficient ducts (Figure 3 Ci, areas 1 and 2) shared a common 11867_11873insC mutation (identified and shown as an insG mutation in the reverse strand sequence, repeated on three independent microdissected areas) that was not present in the distant CCO‐proficient cells (Figure 3Ciii, area 5). The neighbouring CCO‐deficient duct (Figure 3B, Ci) was not related to this; however, it was clonal for a heteroplasmic 957 G > A mutation (Figure 3Cii, areas 3 and 4), and this was also not present in the surrounding CCO‐normal DCIS (Figure 3Ciii, area 5). These data suggest that the rate of clonal expansion is higher in neoplastic breast epithelium than in normal breast epithelium and that multiple competing clones are capable of arising within the same DCIS lesion.

### Clonal populations in normal and neoplastic breast epithelium contain multipotent stem cells

**Figure 4:**
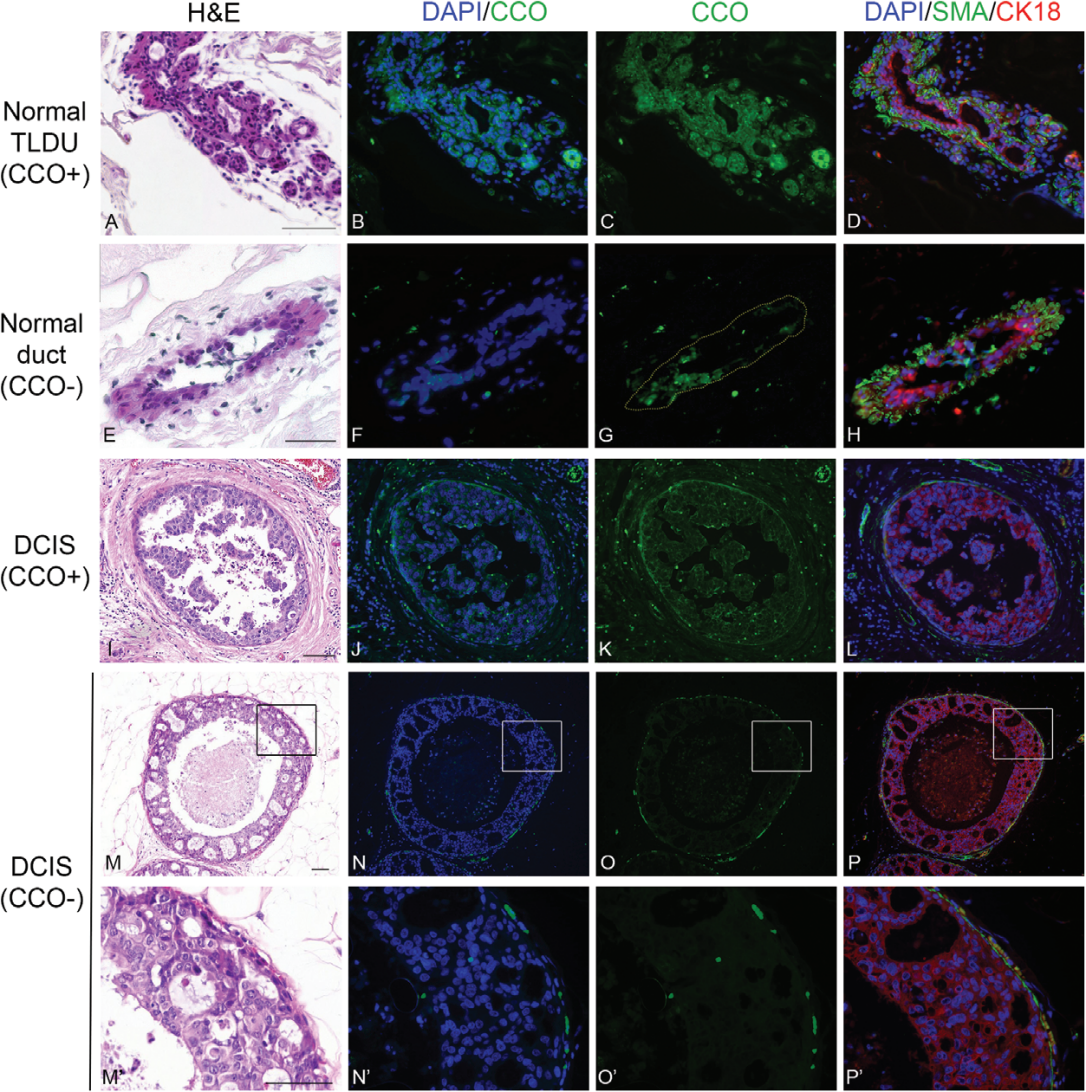
Multipotent stem cells reside within clonal CCO‐negative normal and DCIS ducts. Immunofluorescence staining of serial sections from a normal adult breast (A–H) showed that CCO‐negative areas (lacking green CCO expression, F, G) contained cells positive for markers of luminal cells (CK18, red) and myoepithelial cells (αSMA, green) (H), indicating that multipotent stem cells were present within the CCO‐deficient area and gave rise to the two differentiated cell types. Similarly, immunofluorescence staining of serial sections from a DCIS sample (I–L; M–P and at higher‐power magnification, M‘–P’) showed that CCO‐negative areas (N, O and zoomed areas in N', O') contained cells positive for CK18 and αSMA (green) (P and high power P'), which indicated the presence of multipotent stem cells within the DCIS duct. Scale bar = 75 μm. Haematoxylin and Eosin show tissue structure (A,E,I,M,M').

To investigate whether the clonal CCO‐deficient areas contain multipotent stem cells, we performed fluorescence immunohistochemistry to determine the expression pattern of markers for luminal and myoepithelial cells to seek evidence of multi‐lineage differentiation, the gold standard for stem cell identification 14. Figure 4A–L shows CCO‐deficient epithelial cells in serial sections of normal adult breast co‐localized with αSMA‐positive myoepithelial cells and CK18‐positive luminal epithelial cells. This pattern was observed both in normal adult breast and in DCIS (Figure 4M–P'). While small clusters of fluorescent cells were observed close to the myoepithelial layer in all stained sections (CK18, αSMA, and CCO), they appeared to be autofluorescent blood cells based on morphological features and geographical location (H&E in supplementary material, Figure S1). While we cannot exclude the possibility that these cells could be myoepithelial cells, the vast majority of, if not all, myoepithelial cells were CCO‐negative. This indicates that both the normal and the premalignant mammary epithelium contain multipotent lineages, each maintained by a dedicated population of stem cells.

### Clones restricted to the luminal layer of normal mammary ducts

**Figure 5:**
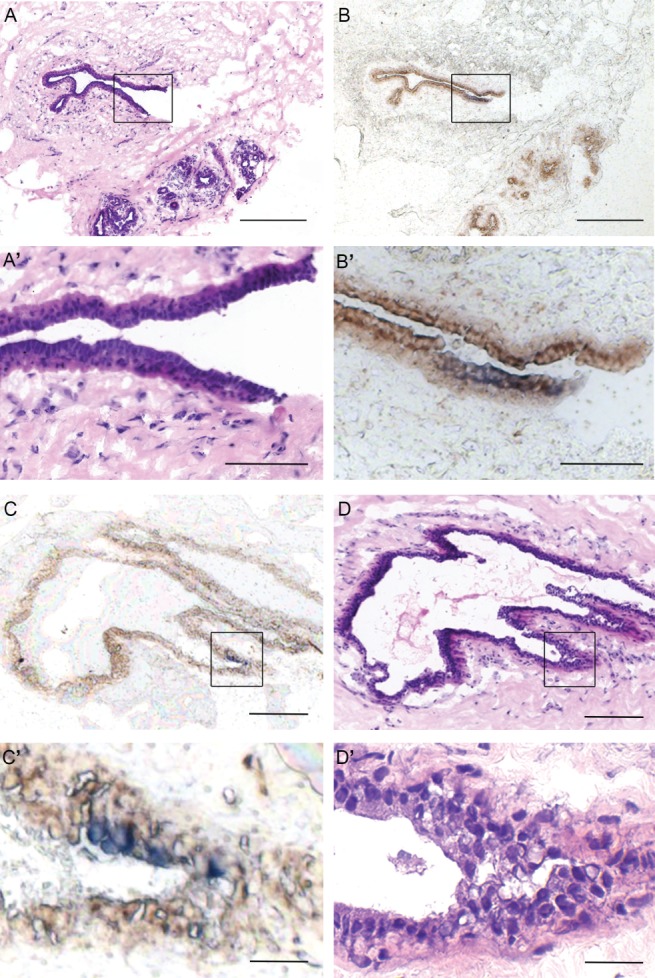
Normal breast contains CCO‐deficient patches of cells restricted to the ductal luminal layer. (A, C) H&E staining showing two ducts in the normal adult breast; high‐power images are shown in A' and C', respectively. (B, D) CCO enzyme histochemistry identified a subset of cells within the luminal layer of the ducts containing blue (CCO‐deficient) cells. High‐power images are shown in B' and D', respectively. Scale bars: A, B = 150 μm; C, D = 300 μm; inset scale bars: A', B', C', D' = 75 μm.

Several studies have argued that mammary stem cells are located in either, or both of, the luminal or myoepithelial layers. A thorough investigation of all CCO‐deficient areas within our cohort of patients revealed a small subset of normal breast samples (2/45; 4.5%) where CCO‐deficient patches were restricted to the luminal cell layer (Figure 5), without involvement of the underlying CCO‐positive myoepithelial layers. We never detected a sample in which CCO deficiency was restricted to the myoepithelial layer, therefore suggesting that each clone is derived from a dedicated progenitor cell located within the luminal epithelial layer.

## Discussion

Lineage tracing in murine models and *in vitro* studies have offered significant insight into the dynamics of stem cells in the mammary gland [1, 28], but the translation of these findings to the normal human breast is uncertain. In this study, using a combination of histological and mitochondrial genetic analysis in human tissues, we obtained evidence that the human adult mammary epithelium is maintained by a population of multipotent stem cells. Areas containing CCO‐deficient cells, which were clonal for mtDNA mutations, were found in the normal adult human mammary epithelium and were shown to contain cells of both luminal and myoepithelial lineages, thus demonstrating that both mammary lineages derive from a long‐lived and multipotent progenitor cell. It has been shown previously that the accrual of a sufficient burden of somatic mutations which result in CCO deficiency may take a considerable period of time (almost 40 years in the human colon) 29. We propose that CCO deficiency originates in the stem cell population, since these are the only long‐lived cells within the epithelium. Consequently, the presence of clonal CCO‐deficient areas in the mammary epithelium that spans both luminal and myoepithelial lineages strongly indicates that a pool of multipotent stem cells maintains the adult human mammary gland.

Areas containing CCO‐deficient cells clonal for mtDNA mutations were also found within TDLUs and along lactiferous ducts, suggesting that a dedicated stem cell population may not be restricted to a specific compartment of the ductal–lobular system. Previous studies, using a variety of putative markers and theoretical models, have proposed that mammary gland stem cells are found at the branch points of side‐ducts 30, in the ducts 10, or in TDLUs, in particular at the edge of growing ductules [11, 31]. A more recent study in the mouse mammary gland excluded the presence of multipotent progenitor cells, but localized unipotent progenitors sporadically in branching ducts or alveoli 32.

Our data do not suggest such a restriction between the regions of the ductal–lobular system, but indicate that multipotent, dividing stem cells are localized along the whole adult mammary epithelium. Furthermore, the presence of multiple CCO‐deficient areas of various sizes within the same duct provides an insight into clonal dynamics and clonal competition in the normal epithelium. Smaller CCO‐deficient areas may represent a new clonal expansion or a clone headed towards extinction, whereas larger CCO‐deficient areas may represent a dominant clone that could eventually lead to a monoclonal conversion of the duct, similarly to the process of crypt purification in the human normal colon 33. We observed mainly areas of CCO deficiency that extended through both layers of the mammary duct: however, in two samples we could detect the presence of CCO‐deficient cells restricted to the luminal layer. This could indicate a differentiation hierarchy relating the two ductal lineages, where the progenitor cells are located in the luminal layer, expand horizontally within this, and only successively derive the myoepithelial layer. We could not detect any case where CCO deficiency was associated uniquely with the myoepithelial layer, supporting previous findings that concluded that the luminal layer is the location of mammary epithelial progenitor cells 2.

CCO‐deficient clonal areas were also detected in ductal carcinoma *in situ* (DCIS), encompassing partial or entire cross‐sections. Although we have no data to show directly the cell of origin of DCIS, we can propose that DCIS originates from stem cells in the luminal layer, as it is likely that this is the cell of origin of clonal expansions within the breast. We observed larger areas of CCO deficiency in DCIS compared with normal breast: if we assume that CCO‐deficient cells represent a snapshot in time of the dynamics of the stem cell populations within the tissue, our findings would indicate an increase in stem cell number in the premalignant lesion.

In summary, we have shown that the adult human breast contains a population of stem cells localized in the whole ductal–lobular system which maintain the normal epithelium by differentiating into both luminal and myoepithelial cells. This architecture is preserved in DCIS but clonal dynamics are altered, and an increase in the size of expanded clones was observed within the premalignant lesions compared with the normal breast.

## Author contributions statement

The authors contributed in the following way: conception and design of the study, experimental work and collection of data, data analysis, interpretation, and manuscript writing: BC and SACM; conducted analysis and interpretation: MJ and NAW; experimental work: EA and GE; provision of study material/patients and manuscript preparation: TM, CHMvD, AMS, MGD, PJT, and LJ. All authors contributed to critical revision of the manuscript and approval of the final version.

**SUPPLEMENTARY MATERIAL ONLINE**

**Supplementary figure legend**

**Figure S1.** Exclusion of autofluorescent erythrocytes from assessment of lineage tracing

## Supporting information


**Supplementary figure legend**

**Figure S1.** Exclusion of autofluorescent erythrocytes from assessment of lineage tracing. (A and C) H&E restain of the same sections from Figure 4N‘and 4P’, here also shown in B and D, respectively, for comparison purposes only. The boxed regions highlight an example of cells that appear to show positive staining for CCO (in B), αSMA and CK18 (in D) in an otherwise CCO‐deficient duct. However, these ‘positive’ cells are likely to be autofluorescent erythrocytes based on the H&E and due to being located externally to the myoepithelial layer. Scale bar = 75 μm.
