# Supplementary material for: Analysis of clonal expansions through the normal and premalignant human breast epithelium reveals the presence of luminal stem cells
Source: J Pathol. 2017 Nov 23;244(1):61–70. doi: 10.1002/path.4989 (PMC5765426; doi:10.1002/path.4989)
Supplement: Supplementary file 1 — Supplementary figure legend [file PATH-244-61-s001.docx]

**Supplementary figure legend**

**Figure S1.** Exclusion of autofluorescent erythrocytes from assessment of lineage tracing. (A and C) H&E restain of the same sections from Figure 4N' and 4P', here also shown in B and D, respectively, for comparison purposes only. The boxed regions highlight an example of cells that appear to show positive staining for CCO (in B), αSMA and CK18 (in D) in an otherwise CCO-deficient duct. However, these ‘positive’ cells are likely to be autofluorescent erythrocytes based on the H&E and due to being located externally to the myoepithelial layer. Scale bar = 75 μm.
